# Comparative Evaluation of Smear Layer Removal in Apical Third Using Four Different Irrigants With Ultrasonic Agitation: An In Vitro Scanning Electron Microscopy (SEM) Analysis

**DOI:** 10.7759/cureus.23142

**Published:** 2022-03-14

**Authors:** Kalaiarasi Murugesan, Sankar Vishwanath, Sadasiva Kadandale, Yashini Thanikachalam, Revathy Parthasarathy, Sangita Ilango

**Affiliations:** 1 Conservative Dentistry and Endodontics, Chettinad Dental College and Research Institute, Chennai, IND

**Keywords:** edta, passive ultrasonic activation, scanning electron microscope, sodium hypochlorite, ozonated water, oxum, smear layer

## Abstract

Aim

This study aims to compare and evaluate the efficacy of four different irrigating solutions like sodium hypochlorite, ethylenediaminetetraacetic (EDTA), Oxum, and ozonated water with ultrasonic agitation in removing the smear layer in the apical third of root canals using Scanning Electron Microscopy (SEM).

Materials and methods

For the study, 50 freshly extracted human mandibular premolars with single well-developed roots without any curvatures were taken. The teeth taken were decoronated to obtain a uniform working length of 15 mm, and the samples were instrumented using a ProTaper Gold rotary file system (Dentsply Maillefer, Ballaigues, Switzerland) up to F2 size, along with irrigation of 1 ml of 3% sodium hypochlorite (NaOCl) in between instrumentation. The samples were randomly divided into five groups with 10 samples each, according to the final irrigant used. Group I-EDTA 17%, Group II-NaOCl 5%, Group III-Oxum, Group IV-ozonated water, and Group V-normal saline. In all groups, ultrasonic agitation of the irrigating solution was performed using a size 20 file, held passively inside the root canal. Then the samples were flushed with distilled water, dried with paper points, split into two halves, and subjected to SEM analysis. SEM images of the apical third region of root samples were taken at 5000X resolution and scored on a scale of 1 to 4.

Results

Statistical analysis was done using one-way ANOVA followed by Tukey’s post hoc test using software version SPSS software version 17.0 (SPSS Inc., Chicago). The results showed that the 17% EDTA group showed the least smear layer scores when compared to other groups with statistical significance. This was followed by the Oxum group and 5% NaOCl group, whereas the ozone water group and saline control group showed the highest smear layer scores.

Conclusion

The present study reveals that the EDTA is the superior irrigant in the elimination of smear layer in root canal treatment. Newer irrigants, such as Oxum, can be used as an alternative to EDTA for smear layer removal while remaining biocompatible with dentin. Whereas ozone can be combined with other irrigants for synergistic action of enhanced antimicrobial property and smear layer elimination in the future.

## Introduction

The smear layer, which is incarnated by the instrumentation process, is found to contain organic and inorganic materials like the odontoblastic process, pulp tissue debris, bacteria, and blood cells [1]. When dentin is cut either by rotary or hand instruments [2,3], the mineralized tissues get crippled to produce a hefty amount of debris, which is made up of a mineralized collagen matrix that spreads over the surface of the dentin to form a smear layer. In order to achieve successful root canal treatment, proper biomechanical preparation, irrigation, disinfection, and obturation are mandatory [4]. Along with this, it is imperative to prepare the root canal in such a way that the filling materials are placed adequately for a competent apical seal. The presence of the smear layer was found to have a nocent effect, as it prevents the penetration of the irrigants and intracanal medicaments into dentinal tubules [5].

Albeit the effect of removing the smear layer on an effective root canal treatment is still debatable, it appears that its removal is preferable to maintain it. Smear layer removal needs a combination of organic component solvents and acids or chelating agents for the removal of inorganic portions [6]. Though umpteen irrigants and irrigating devices are present, the removal of the smear layer remains obscure. Thus, there arises a need to combine irrigants, as the removal of both organic and inorganic debris is strenuous with a single irrigant.

Sodium hypochlorite (NaOCl) acts against an ample spectrum of bacteria and also dissolves vital as well as necrotic tissue. The major disadvantage of NaOCl is that it possesses cytotoxicity when injected into periradicular tissue. Though NaOCl is a more efficient irrigant, it cannot dissolve the inorganic component of the smear layer, thus paving the way for retention of the smear layer during instrumentation [1]. Ethylenediaminetetraacetic acid (EDTA) is a chelating agent that helps in the detachment of biofilms that are adhered to the root canal wall. Several studies have recommended the combination of NaOCl and EDTA, which effectually removes both organic and inorganic debris. Nevertheless, prolonged treatment might result in inadvertent intertubular and peritubular dentin erosion [7], and it is also shown to effectively eliminate the smear layer only in the coronal and middle third, but it is less efficacious in the apical third. Thus, for optimal smear layer removal besides EDTA, newer irrigating agents such as Oxum and ozonated water have been explored as final irrigants in this study.

The aim of the present study is to compare the smear layer removal efficacy of four different irrigating solutions EDTA, NaOCl, Oxum, and ozonated water with ultrasonic agitation in the apical third of root canals using scanning electron microscopy (SEM).

## Materials and methods

Sample selection

After obtaining ethical approval from the Chettinad Academy of Research and Education Institutional Human Ethics Committee for Student Research (IHEC-I/0793/22), 50 freshly extracted human mandibular premolars that were extracted for orthodontic and periodontal reasons were taken for the study.

Inclusion and exclusion criteria

Intact teeth devoid of caries, cracks, and fractures with well-developed roots and closed apices were included. All the teeth were single rooted with single canals (Vertucci’s type I). Dilacerated roots, multirooted teeth, and teeth with multiple canals and fused canals were not included in this study.

Teeth preparation

All the samples were subjected to decoronation to glean a uniform working length of 15 mm using diamond discs. The patency of the canal was established by passing the # 15 K file (Mani Inc., Delhi, India) till it reached the apex. Under magnifying loupes, when the file tip was visible at the apex, the working length was measured at 1 mm short of that length. By using the ProTaper Gold rotary file system (Dentsply Maillefer, Ballaigues, Switzerland), the canals were cleaned and shaped up to F2 size with the manufacturer's recommended speed and torque. In between instrumentation, the canals were irrigated with 1 ml of 3% NaOCl (Prime Dental Products Pvt. Ltd., Thane, India) between each file, while in the control group, normal saline was the sole irrigant.

Group allocation and Irrigation

The tooth samples were divided randomly into five groups, with 10 samples in each group. Group I-Normal Saline (0.9%w/w); Group II-17% EDTA (NEOEDTA liquid, Orikam, Gurugram, India); Group III-5% NaOCl (Chemident, Delhi, India); Group IV-Oxum-Super Oxidized Solution of oxidized water 99.97%w/v + hypochlorous acid 0.006%w/v (Alkem Laboratories Ltd., Mumbai, India); Group V-Ozonated water, freshly prepared using UNO5 oxygen concentrator by infusing oxygen current into 1 L sterile distilled water at 7gh^-1^ (UNO5, UNOSupply, India).

Then each sample was irrigated with 5 ml of each irrigant for 1 minute. The irrigants were delivered into the root canal according to the corresponding groups using a double vented 30 gauge endodontic irrigation needle (RC Twents, Prime dental products Pvt. Ltd., Thane, India). Then in all samples, ultrasonic agitation was performed using a size 20 file (IRR20/21mm, Acteon® IrriSafe™) held passively inside the root canal for 1 minute. Finally, all the root canals were irrigated with 5 ml of distilled water, to get rid of any precipitate. By using sterile paper points (META BioMed, Chungcheongbuk-do, Korea), the canals were blot dried. Diamond discs were used to cut deep grooves, without piercing buccal and lingual surfaces of the root. The roots were truncated with chisel and mallet. One-half of each tooth is selected and prepared for SEM examination.

SEM analysis

The specimens were dried and dehydrated using ascending concentrations of ethyl alcohol (30%-100%). The samples were then air-dried, mounted on metallic stubs, and sputter-coated with gold using an ion sputter coater (JFC 1600 auto fine coater, JEOL, Peabody, USA). These samples were examined under a Scanning Electron Microscope (JSM-6390, JEOL, Japan) for the presence or absence of the smear layer. Photomicrographs at 5000X magnification of root canal walls at the apical third of each specimen were taken.

Scoring criteria

The scoring system described by Prado et al. in 2011 was used to evaluate the degree of smear layer removal [8].

· Score 1: no smear layer and all tubules are clean and open

· Score 2: a few areas covered by smear layer, with most tubules cleaned and opened

· Score 3: smear layer covering almost all the surface, with a few tubules, opened and

· Score 4: smear layer covering all the surfaces.

Two independent observers with blinding performed the scoring, and the average values were taken as final scores.

## Results

**Figure 1:**
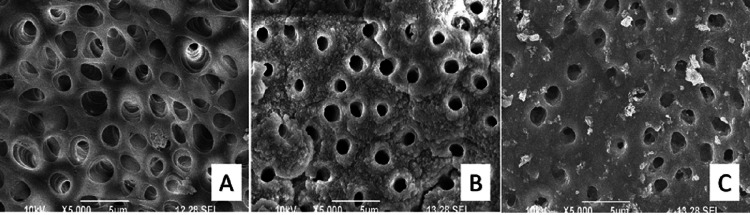
SEM images of experimental groups at 5000X resolution. Group A - EDTA (Ethylenediaminetetraacetic acid) 17%, Group B - NaOCl (Sodium hypochlorite) 5%, Group C - Oxum (Superoxidised solution)

**Figure 2:**
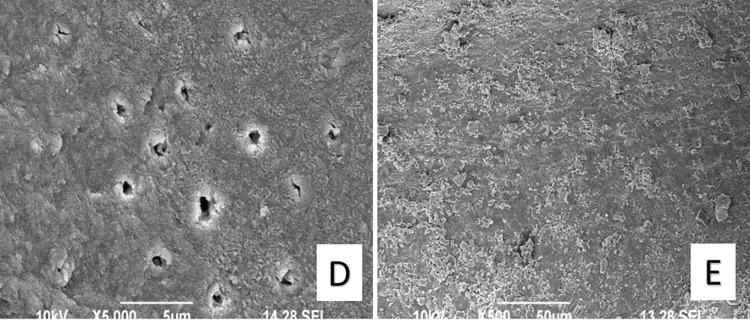
SEM images of experimental groups at 5000X resolution. Group D – Ozonated water (Freshly prepared) Group E – Normal saline (Control group)

The SEM images taken of all the experimental groups at the apical third of the tooth samples are shown in Figures 1, 2.

Data were collected and statistically analyzed using one-way ANOVA followed by Tukey’s post hoc test using software version SPSS 17.0 version (SPSS Inc., Chicago). The significance level is set at a p-value <0.05. Table 1 depicts the smear layer removal scores of various groups at the apical third of root canals with p values.

**Table 1:** Descriptive statistics and intergroup comparison by one-way ANOVA Intergroup comparison by one-way ANOVA shows a highly significant difference (p<0.001) in the smear layer removal between all the experimental irrigation groups. EDTA: Ethylenediaminetetraacetic acid; NaOCl: Sodium hypochlorite; Oxum: Superoxidised solution

Group	Mean	SD	95% Confidence Interval for Mean		F	p-value
			Lower Bound	Upper Bound	37.2	0.001*
Saline	4.0000	.0000	4.0000	4.0000		
EDTA	1.5556	.5270	1.1504	1.9607		
NaOCl	2.5556	.5270	2.1504	2.9607		
Oxum	2.2222	.6666	1.7098	2.7347		
Ozone	3.6667	.5000	3.2823	4.0510		

**Figure 3:**
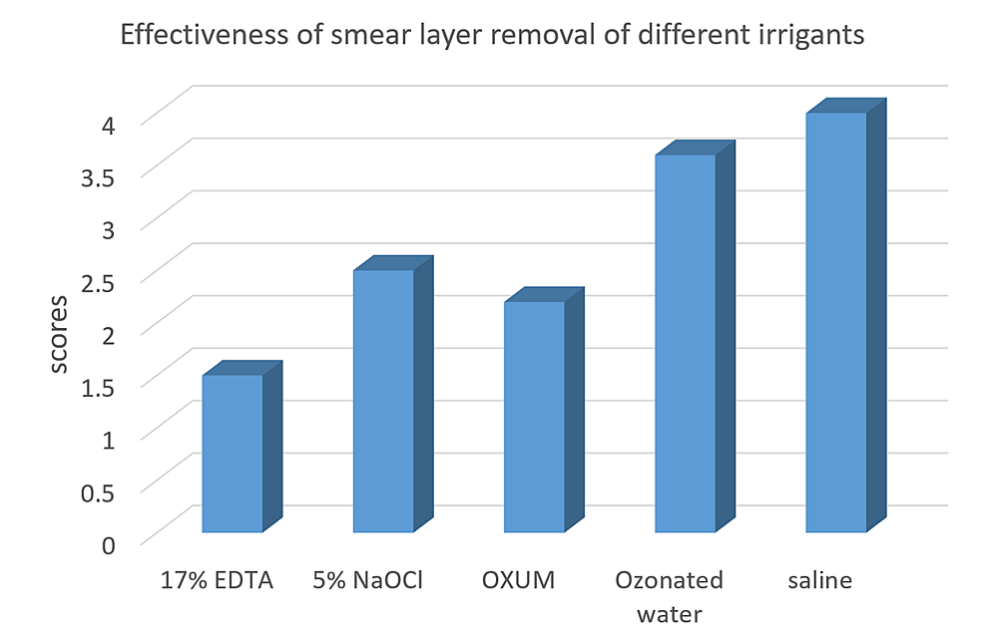
Mean score values of SEM images. The order of smear layer removal effectiveness among all the experimental irrigant groups was in the order of group II˃ group IV˃ group III˃ group V˃ group I (least score to highest score). EDTA: Ethylenediaminetetraacetic acid; NaOCl: Sodium hypochlorite; Oxum: Superoxidised solution

The results of the in-vitro study show a significant difference in the smear layer removal by the different irrigation systems used. The results revealed that Group II (EDTA) was found to be the most effective irrigant with statistical significance in smear layer removal among all five irrigants used. This was followed by the Oxum group and the 5% NaOCl group, whereas the ozone water group and saline control group showed the highest smear layer scores. The ascending order of smear layer removal effectiveness among all the irrigants was in the order of group II˃ group IV˃ group III˃ group V˃ group I (Figure 3).

Table 2 depicts the multiple pairwise comparisons by Tukey’s post hoc test. When the intragroup comparison was done, there was a significant difference between various groups. When pairwise comparison was done, there was no significant difference in the values between saline and ozone, EDTA and Oxum, NaOCl and Oxum.

**Table 2:** Intragroup comparison by Post hoc Tukey’s test Pairwise comparison by Post hoc Tukey's test shows no significant difference in the values between saline and Ozone, EDTA and Oxum, NaOCl and Oxum. EDTA: Ethylenediaminetetraacetic acid; NaOCl: Sodium hypochlorite; Oxum: Superoxidised solution

(I) Group	(J) Group	Mean Difference (I-J)	P value
Saline	EDTA	2.44444*	.000*
	NaOCl	1.44444*	.000*
	Oxum	1.77778*	.000*
	Ozone	.33333	.622
EDTA	Saline	-2.44444*	.000*
	NaOCl	-1.00000*	.001*
	Oxum	-.66667	.053
	Ozone	-2.11111 *	.000*
NaOCl	Saline	-1.44444*	.000*
	EDTA	1.00000*	.001*
	Oxum	.33333	.622
	Ozone	-1.11111 *	.000*
Oxum	Saline	-1.77778*	.000*
	EDTA	.66667	.053
	NaOCl	-.33333	.622
	Ozone	-1.44444*	.000*
Ozone	Saline	-.33333	.622
	EDTA	2.11111 *	.000*
	NaOCl	1.11111 *	.000*
	Oxum	1.44444*	.000*
* Significance value (P<0.05)			

## Discussion

Regardless of the controversy, it is judicious to remove the smear layer in teeth with infected root canals in order to disinfect the entire root canal system. Despite the wide range of irrigants available today, the hunt for the optimal root canal irrigant is a ceaseless problem due to numerous factors like dentine substrate, root canal microbiota, and smear layer, which are difficult to eradicate completely. Since there are so many variables that influence its action, no single irrigant has been found to be effective in eliminating both organic and inorganic debris to date. As a result, eliminating the smear layer demands combining the efficacy of a variety of irrigants, as no one irrigating solution can currently meet all of the ideal criteria [9].

The chemical approach of utilizing chelating agents to remove the smear layer has been the most widely utilized method so far, with ethylenediaminetetraacetic acid (EDTA) being the most widely used agent [1]. The use of EDTA and sodium hypochlorite (NaOCl) alternately has been suggested for effective smear layer removal [10]. However, there is concern that this combined irrigation regimen will result in the intraradicular dentin being eroded inadvertently [11].

In this study, four different irrigating solutions, such as 17% EDTA, Oxum, ozonated water, and 5% sodium hypochlorite, were utilized for the smear layer removal along with ultrasonic agitation. The goal of this study was to assess and compare the efficacy of these irrigants in eradicating the smear layer from the apical portion of the root canal.

When compared to the coronal and middle thirds of the root canal, all of the available irrigants were found to be less successful in penetrating the apical third, which is due to the stagnation plane of residual fluid in the apical third [12].

Because of gas-particle entrapment and complicated anatomy, traditional irrigation systems typically fail to deliver to the apical third. The apical third can thus be appropriately accessed by ultrasonic activation and/or the addition of a detergent that lowers surface tension. Mechanical stimulation efficiently reduces the smear layer in the apical third by eliminating air bubbles that impede penetration [13].

In this study, 30 gauge double-side vented irrigation needles were used, which can creep into the apical one-third because of their small-bore size and also increase the effective contact of the irrigant to the canal wall for smear layer removal and intercept the vigorous passage of irrigants through the apical foramen [14].

The results of the present study, comparing EDTA, NaOCl, Oxum, and ozone for the removal of organic and inorganic components of the smear layer, revealed that 17% EDTA was found to be the most effective, followed by Oxum. Results in intragroup comparison revealed that there was no significant difference in the values between saline and ozone, EDTA and Oxum, NaOCl and Oxum.

In this study, when EDTA alone with ultrasonic agitation was used to irrigate the canal, it entirely eliminated the smear layer, indicating that ultrasonic agitation promotes clearance of organic debris in addition to its inherent property of smear layer removal by EDTA [14,15]. This is consistent with Wu et al.’s findings that 17% EDTA was more successful in eliminating the smear layer than other irrigants such as 20 percent citric acid and Biopure MTAD [16].

Though the substantial body of research supported the use of 17% EDTA in combination with NaOCl to remove the smear layer, the majority of these studies contradicted the use of NaOCl alone to remove the smear layer. In the current study, smear layer removal utilising 5% NaOCl and passive ultrasonic irrigation was equivalent to that of EDTA and oxum. This is in accordance with Cameron et al. in 1983, who stated that NaOCl for one minute with ultrasonic activation eliminated the overlying superficial smear layer but left the dentinal tubules sealed off [17].

NaOCl, which has a good antibacterial effect when coupled with ultrasonic activation, also increases the debris/smear layer removal by producing shear stress in the smear layer's inorganic particles by acoustic streaming, making it easier to remove [18,19].

In the current study, EDTA 17% showed more damaged dentinal tubules and intertubular dentin. The organic part was removed using 5% NaOCl, while the inorganic half of the smear layer was left intact. Both organic and inorganic smear layers were efficiently removed by Oxum without altering the appearance of the dentinal surface.

Oxum is a super oxidised aqueous solution that is electrochemically processed and made from pure solutions that are high in reactive oxygen species. Due to its neutral pH and prolonged half-life, super oxidised water is an FDA-approved stable irrigant utilised for wound care therapy. Oxum is a hypotonic solution composed primarily of sodium hypochlorite, hypochlorous acid, ozone, and hydrogen peroxide. It is a well-known bactericidal, fungicidal, virucidal, and sporicidal substance that releases free radicals upon electrolysis, which rapidly react and denature proteins in the bacterial cell wall [20].

In the present study, oxum showed better results in smear layer removal than EDTA [21]. The smear layer was removed in large areas, leaving the collagen fibres intact and exposed with reduced erosion. This is consistent with findings from Mensudar et al. in 2016, they found that smear layer removal was greatest in the coronal third compared to the middle and apical thirds, and it eliminated the smear layer in larger regions with less erosion [22].

Oxum also has the benefit of being biocompatible with the host tissues. Because multicellular organisms are not harmed by the irrigant's osmolarity variations, the irrigant solely damages the cell membrane of single-cell organisms and denatures bacterial proteins.

Much recent research has demonstrated the advantages of using ozone in root canal disinfection. When compared to other ozonated products, ozonated water has been shown to have high efficacy and superior bactericidal action. Ozonated water has been proven to be equally effective as NaOCl as a root canal irrigant in anti-bacterial efficacy. Furthermore, they are extremely biocompatible with tissues and do not appear to change the enamel or dentin characteristics [23]. It has also been claimed that ozone could be used to open up dentinal tubules for restorative procedures [24].

Thus, in this study, ozone was utilised in combination with ultrasonic activation to remove the smear layer, and the results show that ozone water has an insignificant smear layer removal ability when compared to the other irrigants examined. In the future, a synergistic action of enhanced antimicrobial properties and smear layer elimination might be attempted by combining ozone with other irrigants having smear layer removal properties, such as mild acids [25,26].

Within the limitations of this study, Oxum is equivalent to EDTA in eliminating the smear layer. The presence of blood, tissue remnants, and a heap variable, however, may affect the effects of irrigants in the root canal system, so this study needs to be replicated in vivo for further validation. In addition, removing the smear layer in curved canals is more difficult and perplexing. More research into long-term clinical investigations is required to validate these findings and assess their consistency in treatment outcomes.

## Conclusions

Within the limitations of the study, it is concluded that 17% EDTA is the superior irrigant in the elimination of the smear layer in root canal treatment. Organic material was eliminated with 5% NaOCl, whereas the inorganic half of the smear layer remained intact. Newer irrigants, such as Oxum, can be used as an alternative to EDTA for smear layer removal while remaining biocompatible with dentin. Ozone can be combined with other irrigants for synergistic action of enhanced antimicrobial property and smear layer elimination in the future.
